# Discriminating Bio-aerosols from Non-Bio-aerosols in Real-Time by Pump-Probe Spectroscopy

**DOI:** 10.1038/srep33157

**Published:** 2016-09-13

**Authors:** Gustavo Sousa, Geoffrey Gaulier, Luigi Bonacina, Jean-Pierre Wolf

**Affiliations:** 1 Université de Genève, GAP-Biophotonics, 22 chemin de Pinchat, Carouge, 1211 Geneva 4, Switzerland

## Abstract

The optical identification of bioaerosols in the atmosphere and its discrimination against combustion related particles is a major issue for real-time, field compatible instruments. In the present paper, we show that by embedding advanced pump-probe depletion spectroscopy schemes in a portable instrument, it is possible to discriminate amino acid containing airborne particles (bacteria, humic particles, etc.) from poly-cyclic aromatic hydrocarbon containing combustion particles (Diesel droplets, soot, vehicle exhausts) with high selectivity. Our real-time, multi-modal device provides, in addition to the pump-probe depletion information, fluorescence spectra (over 32 channels), fluorescence lifetime and Mie scattering patterns of each individually flowing particle in the probed air.

The real-time detection and identification of bioaerosols in the atmosphere, such as airborne bacteria and pollens, is an active domain of research. In particular, several optical systems^1^,^2^,^3^,^4^,^5^,^6^,^7^,^8^,^9^,^10^,^11^,^12^, based on fluorescence and/or elastic scattering have been developed to distinguish bio-aerosols (bacteria, pollen, etc.) from non-bio-aerosols, like combustion related hydrocarbons and soot. The most advanced approaches interrogate individual aerosol particles, whose fluorescence is spectrally resolved and analyzed in real time. Dual wavelength excitation (for instance 263 nm and 351 nm) of single particles has emerged as an additional tool for improving the measurement selectivity and therefore reducing false alarms^13^,^14^,^15^,^16^. Some extensive studies have been recently performed in order to investigate the limits of linear UV-Visible spectroscopy (using extended 2D excitation-fluorescence spectra) for tearing apart the signatures of fluorescent aerosols in the air^17^,^18^. Alternately, non-linear spectroscopic techniques such as harmonic-19 and plasma-generation^20^,^21^, and multi-photon excited fluorescence^11^,^22^,^23^ have emerged as promising alternatives, but they appeared difficult to translate into field-compatible devices. In particular, femtosecond pump-probe depletion spectroscopy^24^,^25^ and quantum control^25^,^26^,^27^,^28^ demonstrated their ability for discriminating bioparticles from non-bioparticles in solution in the laboratory, as well as identifying biomolecules with strongly overlapping spectra. In this latter case, the underlying mechanisms allowing fluorescence depletion for amino acids (AA) and absence of fluorescence depletion for other organic compounds relies on the difference in excited state absorption (ESA) characteristics. As outlined in the scheme on the right in Fig. 1, from the intermediate excited state *S*_1_ (populated by the pump photon), the probe photon re-excites the molecules in some upper lying states *S*_*n*_, which are likely to autoionize or dissociate, yielding *S*_1_ depopulation and fluorescence depletion. This is the case of AA, but not the case of other aromatic hydrocarbons, in which either (1) the transition moment *S*_1_ → *S*_*n*_ is small or (2) *S*_*n*_ decays non-radiatively to *S*_1_, leading to the same final population in *S*_1_, and thus, the same fluorescence as for the UV excitation only.

Although promising, most of these non-linear spectroscopic methods relied on complex femtosecond laser systems, which prevented their transferability for field measurements. In the present paper we demonstrate that pump-probe depletion discrimination concepts can be transferred to practical applications by using nanosecond 263 nm pump and 527 nm probe lasers; fluorescence depletion by a 527 nm ns pulse is indeed found efficient for AA containing bioaerosols (bacteria, humic acid, protein containing droplets, etc.), and almost effect less for combustion related organics (Polycyclic aromatic hydrocarbon (PAHs), diesel droplets, soot, vehicle exhaust emission, etc.). On this basis, we developed a portable instrument, bearing multi-modal capabilities: optical scattering in the near infrared (NIR), UV laser-induced fluorescence spectrally resolved over 32 spectral channels, fluorescence lifetime, and a disruptive pump-probe depletion methodology. All these optical data are recorded in real-time and on each individually flowing aerosol particle. We demonstrate, in particular, the unique advantages of pump-probe depletion spectroscopy for discriminating bio- from non-bio-aerosols.

## Experimental Set-up

**Figure 1:**
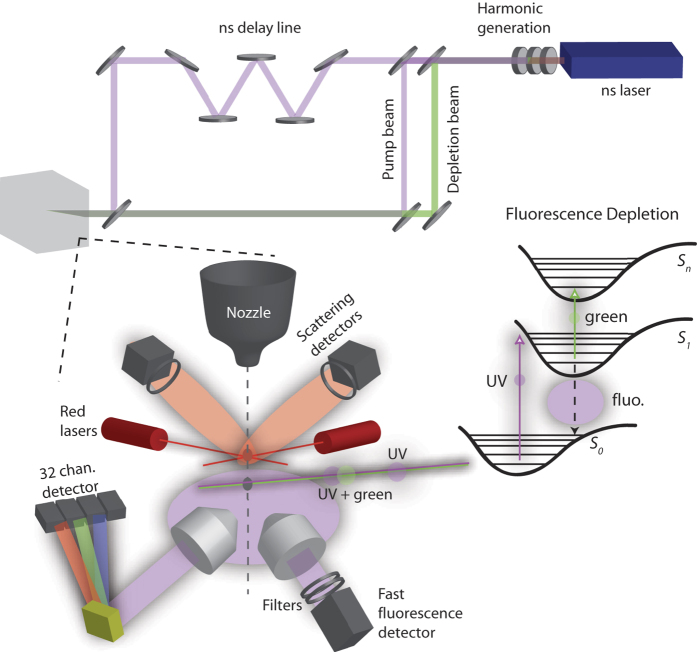
Experimental set-up and fluorescence depletion scheme.

The experiment was inspired by the successful design pioneered about 15 years ago by R.K. Chang at Yale together with the ARL^7^. This single-particle fluorescence spectrometer was then further developed in our group, and specifically modified here for implementing the 263 nm pump - 527 nm probe concept. More precisely, as reported in Fig. 1, the air is sampled at 60 L/min, concentrated by a 10× aerodynamic lens, and then focused by an optimally-designed sheath nozzle in a narrow stream of 500 *μ*m diameter^29^. The concentrator was not used in this work and therefore the total flow was limited to 6 L/min composed by 3 L/min sample flow and 3 l/min sheath flow. The stream then crosses two infrared diode lasers for Mie scattering measurements (690 nm), which allows the determination of the optical size of each particle. Scattering signals are also used to trigger a pulsed DPSS Nd:YLF laser, which provides both 10 *μ*J at 263 nm and 225 *μ*J at 527 nm, with a maximum repetition rate of 1 kHz. The UV polarization is set parallel to the visible one by a half wave plate. The visible radiation is separated by a dichroic mirror, which transmits 263 nm and reflects 527 nm. The UV is split in two arms by a partially reflective mirror (R 50% at 263 nm). Half of the beam is recombined with the visible radiation by a dichroic mirror. The two colors are thus temporally overlapped (optimally, the visible should be slightly shifted by 1–2 ns). The second part of the UV beam is temporally shifted by typically 20 ns by a delay line formed by a set of HR (high reflectivity) UV mirrors. The delayed UV beam is then recombined to the other two overlapped beams by another 50% reflectivity UV mirror, which transmits also the visible. The sequence of laser pulses is then focused onto the aerosol flow in the measuring chamber. A dedicated channel is used for the fluorescence depletion/lifetime measurement, constituted by a collecting lens, a rejection filter at 527 nm, a pass-band filter, and a fast photomultiplier tube (PMT). The PMT transient signal is analyzed using a 300 MHz oscilloscope. Simultaneously, the fluorescence is collected by a separate Schwarzschild reflective objective and spectrally analyzed in 32-channels from 300 nm to 500 nm. This spectrometer is based on a multi-anode photomultiplier (Hamamatsu H7260-03) with home-designed acquisition electronics, as described previously^12^,^29^. The whole instrument is compact (*L* × *l* × *h* = 60 cm × 60 cm × 40 cm) and rugged, compatible with field measurements. The maximal detection rate is approximately 10^4^ events/min.

## Results and Discussion

Several types of aerosols were analyzed in order to assess the discrimination ability offered by the fluorescence spectrum/lifetime/pump-probe depletion approach. A TSI aerosol generator (Model 3076) and a nebulizer were used to inject reference material, like *Enterococcus* bacteria, diesel droplets, tryptophan particles and humic particles, while the exhausts of two different Diesel cars were used for analyzing non-bioparticles emitted by combustion in actual conditions. The typical size distributions estimated by the scattering signals from the NIR lasers are provided in the Supplementary Fig. S1. Note that at this stage no special effort was paid to detect single bacteria, and we mostly observed small aggregates due to the sample preparation process.

**Figure 2:**
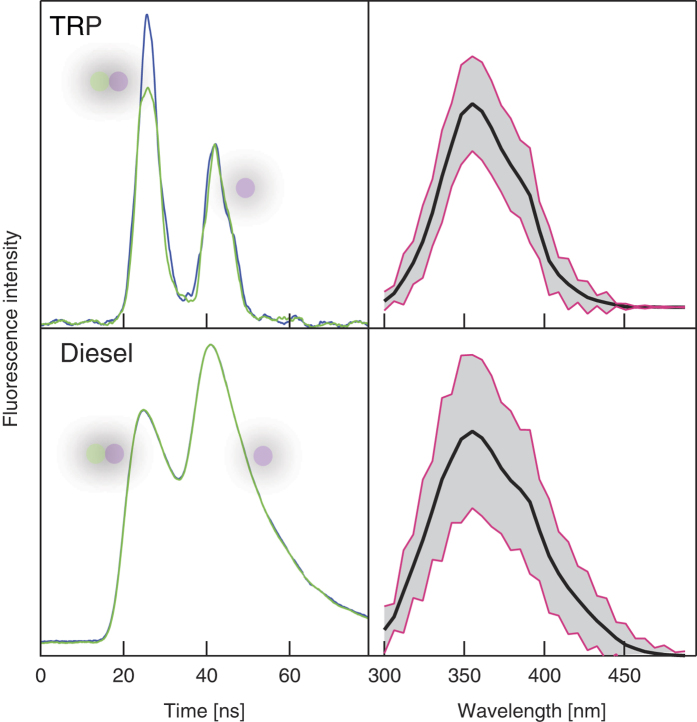
Left, fluorescence depletion response. Averaged oscilloscope traces acquired by the fast detector in Fig. 1 in absence (purple line) and presence (green line) of the depletion pulse. Note that the depletion pulse is temporally superposed with the UV pump pulse only for the first peak, while the second peak is acquired as a reference for fluorescence intensity for the same aerosol particle. *Right, averaged fluorescence spectra.* The greyed regions cover an area of two standard deviations around the data points as obtained for approximately 10^3^ detection events.

**Figure 3:**
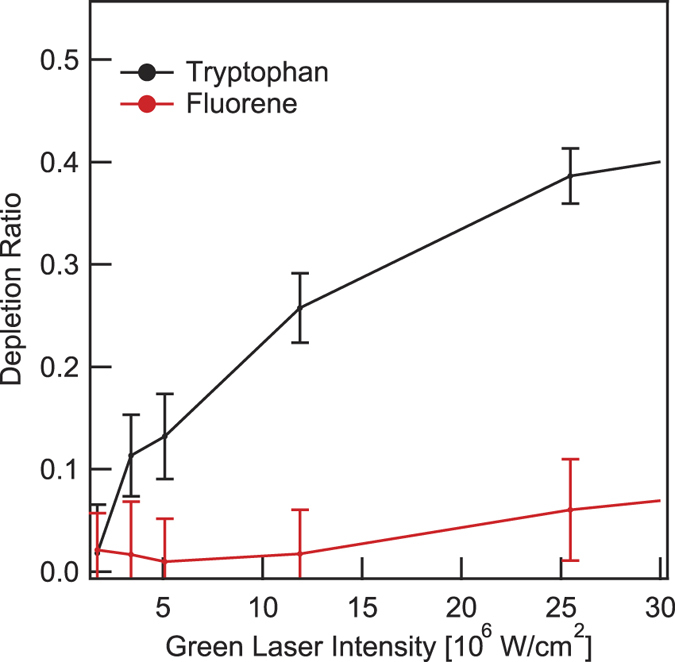
Intensity dependence of fluorescence depletion. For the depletion ratio, value 1 corresponds to total depletion and 0 to absence of any effect on the fluorescence intensity by the green laser.

Figure 2 shows typical signals, simultaneously recorded on the pump-probe/lifetime channel (left column) and on the spectrally resolved fluorescence spectrometer channel (right column). These traces consist of the cumulative signals from 10^3^ individual particles for two species: tryptophan particles and Diesel drops. Large intensity fluctuations are recorded between the different events (greyed regions in the spectral plots), but the overall spectral signature is preserved for both species. However, the plots also evidence the fact that linear fluorescence spectroscopy is unable to discriminate between the AA fluorescence and the different PAH fluorescence in the Diesel drop, as the bands’ widths and peak positions are very similar. The discrimination is provided by the time resolved, pump-probe signals, on the left column. As can be seen from the upper left quadrant, the tryptophan particle’s fluorescence is depleted by typically 20% by the 527 nm pulse, the second pulse in time serving as a reference. In contrast, no depletion is observed for the Diesel droplets. Additionally, the time resolved fluorescence (estimated by fitting the PMT oscilloscope trace by a Gaussian curve) displays a short, <5 ns lifetime (limited by the instrumental response), for tryptophan and bacteria, as previously reported^30^,^31^,^32^. Much longer, 16 ns average, for the Diesel mixture. This longer fluorescence lifetime for different PAHs, ranging from 4 ns to 36 ns, was already studied in the literature^33^, in line with our present observations. A legitimate question is then whether lifetime and depletion ratios are connected, as both observables are related to excited state dynamics. For instance, a short lifetime PAH, like fluorene (5.9 ns), will hardly be discriminated from tryptophan (3.7 ns)^30^ by its lifetime. In order to assess whether, in these conditions, pump-probe spectroscopy would be efficiently discriminative, we measured the fluorescence depletion ratio of fluorene, as a function of the 527 nm probe laser intensity. The results, shown in Fig. 3, demonstrates the unique capability of pump-probe depletion as compared to spectrally resolved or fluorescence lifetime approaches. The fluorescence depletion *D*, is defined as *D* = 1 − *I*_*d*_/*I*_*u*_, where *I*_*u*_ is the undepleted fluorescence intensity (without probe) and *I*_*d*_ the depleted fluorescence intensity (with probe), respectively. Fluorescence depletion *D* is clearly above noise level already at low probe intensities and it rises until 0.5 for tryptophan, while *D* always remains almost negligible for fluorene. This demonstrates that even a PAH with similar spectrum and similar lifetime as an AA can be discriminated by the present pump-probe depletion method. This suggests that the depletion efficiency mainly relies on the ESA cross-section to *S*_*n*_ at 527 nm, or that the relaxation pathways from *S*_*n*_ are intrinsically different between AAs and PAHs (for instance the branching towards vibrational non-radiative cascading down to *S*_1_).

**Figure 4:**
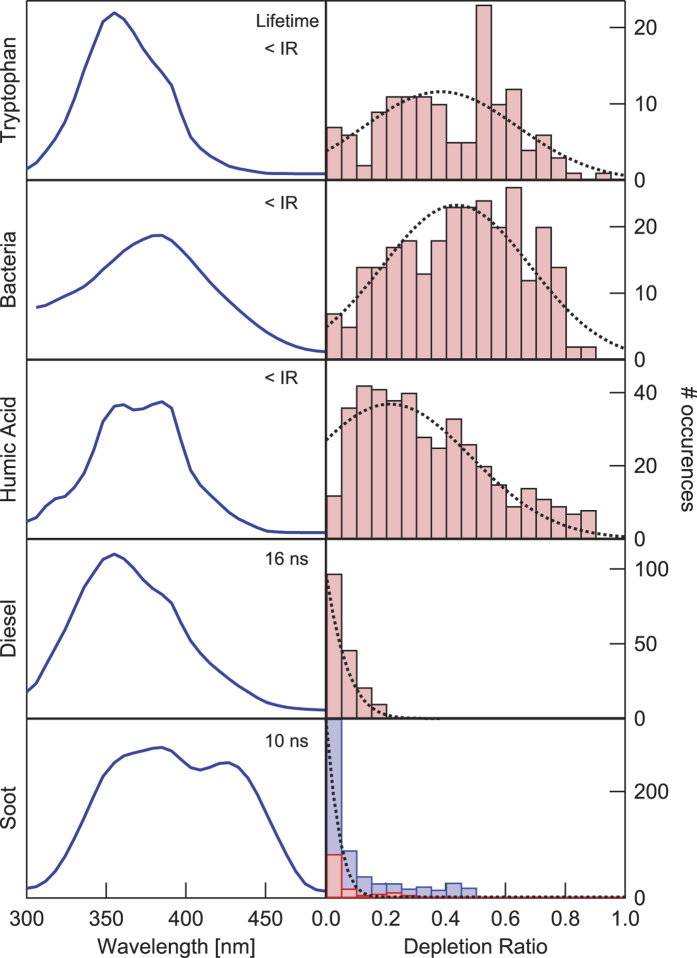
Left, averaged fluorescence spectra and measured lifetimes for several different aerosol particles. <IR indicates that the measured lifetime is limited by the instrumental response (~5 ns). *Right, distribution of depletion events.* In these histograms, 1 corresponds to total depletion and 0 to absence of effect on the fluorescence intensity. For soot we present two set of data for depletion (red and blue) corresponding to two different Diesel vehicles. The distributions are fitted by a Gaussian function.

In order to evaluate the performance of the present approach, we analysed different aerosol particles present in the air, including mineral dust, bacteria (*Enterococcus*), humic particles, liquid organic droplets, and carbonaceous particles (soot) emitted by 2 different Diesel vehicles. As can be seen from Fig. 4, some difference exist in the recorded fluorescence spectra of the species, which can be exploited for helping identifying them. The spectral overlap is, however, significant, so that spectrally resolved data are not sufficient for discriminating the bio- from the non-bio- particles. In contrast, pump-probe depletion clearly categorizes the two types of aerosols, based on their content in AAs or not. So, Diesel droplets and soot from two 15 years old vehicles, one with a badly working engine (blue bars) producing large soot clusters, another one with engine in order (red bars) producing smaller particles exhibit negligible depletion ratios, while bacteria and humic particles exhibit average depletion ratios between 0.3 and 0.5. Therefore, setting an arbitrary threshold for *D* at 0.15 (at this particular probe intensity) substantially discriminates bio-aerosols from non-bio-aerosols, yielding 85% correct detections in the case of bacteria, 100% for Diesel and 80 and 100% for the two data-sets of soot, respectively. Note that this discrimination levels are computed applying exclusively this threshold-based classification, while they can be improved by a multi-parametetric analysis which includes, for example, information on size, signal intensity, etc. As a side note, we remark that depending on the application, the threshold *D* can be finely adjusted. For instance, when monitoring pathogens in public indoor areas like airports, it might be wiser to limit false positive rate from soot to avoid false alarms.

## Conclusions

Fluorescence depletion is generic for AAs^25^,^28^, peptides^34^, and flavin^27^ containing particles. For instance, femtosecond pump-probe depeletion was investigated for different types of bacteria^25^ (*Bacillus Subtilis, Escherichia Coli, Enterococcus*, etc.) and very similar depletion ratios were found for all of them. This ensures that most of, if not all, bioaerosols will be identified as such by the pump-probe depletion methodology. However, this also demonstrates that the same methodology is unable to discriminate among different bioaerosols. A first attractive field experiment would be to assess in real-time the fraction of bioaerosols from traffic related carbonaceous emissions at the periphery of a large urban area or at rural sites close to a highway or a road tunnel. AAs are indeed identified as excellent bioaerosols proxy in urban environments^35^. A more technical, but attractive, outcome in the case of urban areas, would be to assess the fraction of bioaerosols in each of the different “spectra clusters” that have been often identified by self-referencing classification in the studies using spectrally resolved fluorescence measurements of individual particles^36^,^37^,^38^,^39^. This would allow to revisit these data with a new point of view, and ultimately extract more information about the sources that generated these generic clusters.

## Additional Information

**How to cite this article**: Sousa, G. *et al*. Discriminating Bio-aerosols from Non-Bio-aerosols in Real-Time by Pump-Probe Spectroscopy. *Sci. Rep.* **6**, 33157; doi: 10.1038/srep33157 (2016).

## Supplementary Material

Supplementary Information

## References

[b1] Pan Y. L. . Single-particle fluorescence spectrometer for ambient aerosols. Aerosol Sci. Tech. 37, 628–639 (2003).

[b2] Reyes F. . Bio-aerosol fluorescence sensor. Field Anal. Chem. Tech. 3, 240–248 (1999).

[b3] Eversole J. . Continuous bioaerosol monitoring using uv excitation fluorescence: outdoor test results*. Field Anal. Chem. Tech. 5, 205–212 (2001).

[b4] Luoma G. A., Cherrier P. P. & Retfalvi L. A. Real-time warning of biological-agent attacks with the canadian integrated biochemical agent detection system ii (cibads ii). Field Anal. Chem. Tech. 3, 260–273 (1999).

[b5] Pan Y.-L., Aptowicz K. B., Chang R. K., Hart M. & Eversole J. D. Characterizing and monitoring respiratory aerosols by light scattering. Opt. Lett. 28, 589–591 (2003). 12703909 10.1364/ol.28.000589

[b6] Kaye P., Hirst E. & Wang-Thomas Z. Neural-network-based spatial light-scattering instrument for hazardous airborne fiber detection. Appl. Optics 36, 6149–6156 (1997). 10.1364/ao.36.006149 18259463

[b7] Pinnick R. G. . Real-time measurement of fluorescence spectra from single airborne biological particles. Field Anal. Chem. Tech. 3, 221–239 (1999).

[b8] Kaye P. . Single particle multichannel bio-aerosol fluorescence sensor. Opt. Express 13, 3583–3593 (2005). 19495264 10.1364/opex.13.003583

[b9] Stanley W. R. . Continuous bioaerosol monitoring in a tropical environment using a uv fluorescence particle spectrometer. Atmos. Sci. Lett. 12, 195–199 (2011).

[b10] Robinson N. H. . Cluster analysis of wibs single-particle bioaerosol data. *Atmo. Meas. Tech.* (2013).

[b11] Kiselev D., Bonacina L. & Wolf J.-P. Individual bioaerosol particle discrimination by multi-photon excited fluorescence. Opt. Express 19, 24516–24521 (2011). 22109478 10.1364/OE.19.024516

[b12] Cobler P. . High-speed, high-sensitivity aerosol fluorescence spectrum detection using a 32-anode photomultiplier tube detector. Rev. Sci. Instrum. 72, 1831–1836 (2001).

[b13] Pan Y.-L. . Fluorescence spectra of atmospheric aerosol particles measured using one or two excitation wavelengths: Comparison of classification schemes employing different emission and scattering results. Opt. Express 18, 12436–12457 (2010). 20588371 10.1364/OE.18.012436

[b14] Pan Y.-L. . Dual-excitation-wavelength fluorescence spectra and elastic scattering for differentiation of single airborne pollen and fungal particles. Atmos. Environ. 45, 1555–1563 (2011).

[b15] Sivaprakasam V., Huston A., Scotto C. & Eversole J. Multiple uv wavelength excitation and fluorescence of bioaerosols. Opt. Express 12, 4457–4466 (2004). 19483996 10.1364/opex.12.004457

[b16] Sivaprakasam V., Lin H.-B., Huston A. L. & Eversole J. D. Spectral characterization of biological aerosol particles using two-wavelength excited laser-induced fluorescence and elastic scattering measurements. Opt. Express 19, 6191–6208 (2011). 21451645 10.1364/OE.19.006191

[b17] Pan Y.-L. . Spectrally-resolved fluorescence cross sections of aerosolized biological live agents and simulants using five excitation wavelengths in a bsl-3 laboratory. Opt. Express 22, 8165–8189 (2014). 24718194 10.1364/OE.22.008165

[b18] Pöhlker C., Huffman J. & Pöschl U. Autofluorescence of atmospheric bioaerosols fluorescent biomolecules and potential interferences. Atmos. Meas. Tech. 5, 37–71 (2012).

[b19] Kasparian J. . Angular dependences of third harmonic generation from microdroplets. Phys. Rev. Lett. 78, 2952 (1997).

[b20] Baudelet M. . Femtosecond time-resolved laser-induced breakdown spectroscopy for detection and identification of bacteria: A comparison to the nanosecond regime. J. Appl. Phys. 99, 084701 (2006).

[b21] Saari S. . Identification of single microbial particles using electro-dynamic balance assisted laser-induced breakdown and fluorescence spectroscopy. Aerosol Sci. Tech. 50, 126–132 (2016).

[b22] Hill S. C. . Enhanced backward-directed multiphoton-excited fluorescence from dielectric microcavities. Phys. Rev. Lett. 85, 54 (2000). 10991157 10.1103/PhysRevLett.85.54

[b23] Sivaprakasam V., Lou J. W., Currie M. & Eversole J. D. Two-photon excited fluorescence from biological aerosol particles. J. Quant. Spectr. RA 112, 1511–1517 (2011).

[b24] Courvoisier F. . Femtosecond laser pulses distinguish bacteria from background urban aerosols. Appl. Phys. Lett. 87, 063901 (2005).

[b25] Courvoisier F. . Discriminating bacteria from other atmospheric particles using femtosecond molecular dynamics. J. Photoch. Photobio. A 180, 300–306 (2006).

[b26] Brixner T., Damrauer N., Niklaus P. & Gerber G. Photoselective adaptive femtosecond quantum control in the liquid phase. Nature 414, 57–60 (2001). 11689940 10.1038/35102037

[b27] Roth M. . Quantum control of tightly competitive product channels. Phys. Rev. Lett. 102, 253001 (2009). 19659071 10.1103/PhysRevLett.102.253001

[b28] Rondi A., Bonacina L., Trisorio A., Hauri C. & Wolf J.-P. Coherent manipulation of free amino acids fluorescence. Phys. Chem. Chem. Phys. 14, 9317–9322 (2012). 22395710 10.1039/c2cp23357f

[b29] Kiselev D., Bonacina L. & Wolf J.-P. A flash-lamp based device for fluorescence detection and identification of individual pollen grains. Rev. Sci. Instrum. 84, 033302 (2013). 23556810 10.1063/1.4793792

[b30] Xu J. & Knutson J. R. Quasi-static self-quenching of trp-x and x-trp dipeptides in water: ultrafast fluorescence decay. J. Phys. Chem. B 113, 12084–12089 (2009). 19708715 10.1021/jp903078x PMC3412955

[b31] Alimova A. . Native fluorescence changes induced by bactericidal agents. IEEE. Sens. J. 5, 704–711 (2005).

[b32] Berezin M. Y. & Achilefu S. Fluorescence lifetime measurements and biological imaging. Chem. Rev. 110, 2641–2684 (2010). 20356094 10.1021/cr900343z PMC2924670

[b33] Dvorak M. A., Oswald G. A., Van Benthem M. H. & Gillispie G. D. On-the-fly fluorescence lifetime determination with total emission detection in hplc. Anal. Chem. 69, 3458–3464 (1997). 21639268 10.1021/ac961095j

[b34] Afonina S. . Discriminability of tryptophan containing dipeptides using quantum control. Appl. Phys. B-Lasers O 111, 541–549 (2013).

[b35] Abe R. Y., Akutsu Y. & Kagemoto H. Protein amino acids as markers for biological sources in urban aerosols. Environ. Chem. Lett. 1–7 (2015).

[b36] Pinnick R. . Fluorescence spectra and elastic scattering characteristics of atmospheric aerosol in las cruces, new mexico, usa: Variability of concentrations and possible constituents and sources of particles in various spectral clusters. Atmos. Environ. 65, 195–204 (2013).

[b37] Pan Y.-L., Pinnick R. G., Hill S. C. & Chang R. K. Particle-fluorescence spectrometer for real-time single-particle measurements of atmospheric organic carbon and biological aerosol. Environ. Sci. Technol. 43, 429–434 (2008). 19238975 10.1021/es801544y

[b38] Pan Y.-L., Pinnick R. G., Hill S. C., Rosen J. M. & Chang R. K. Single-particle laser-induced-fluorescence spectra of biological and other organic-carbon aerosols in the atmosphere: Measurements at new haven, connecticut, and las cruces, new mexico. J. Geophys. Res. 112 (2007).

[b39] Pinnick R. G., Hill S. C., Pan Y.-L. & Chang R. K. Fluorescence spectra of atmospheric aerosol at Adelphi, Maryland, USA: measurement and classification of single particles containing organic carbon. Atmos. Environ. 38, 1657–1672 (2004).

