# Supplementary material for: Discriminating Bio-aerosols from Non-Bio-aerosols in Real-Time by Pump-Probe Spectroscopy
Source: Sci Rep. 2016 Sep 13;6:33157. doi: 10.1038/srep33157 (PMC5020503; doi:10.1038/srep33157)
Supplement: Supplementary Information [file srep33157-s1.pdf]

Supplementary material

Discriminating Bio-Aerosols from Non-Bio-Aerosols in Real-Time by Pump-Probe Spectroscopy

GUSTAVO SOUSA, GEOFFREY GAULIER, LUIGI BONACINA*, AND JEAN-PIERRE WOLF

[1] *Université de Genève, GAP-Biophotonics, 22 chemin de Pinchat, Carouge, 1211 Geneva 4, Switzerland*
**Corresponding author: luigi.bonacina@unige.ch*

1- Size distribution

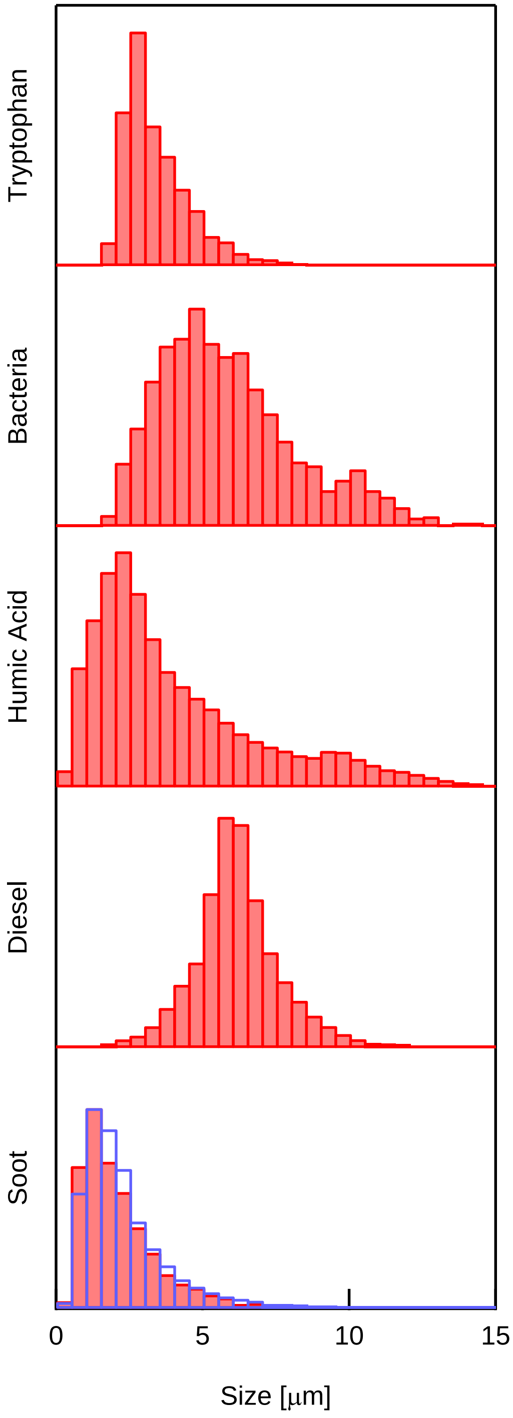

Figure S1. Size distribution of the aerosol samples investigated determined by the scattering signal of NIR lasers..
